# Supplementary material for: The light at the end of the tunnel? A systematic review of higher education student experiences of hope
Source: PLoS One. 2024 Jun 17;19(6):e0304596. doi: 10.1371/journal.pone.0304596 (PMC11182537; doi:10.1371/journal.pone.0304596)
Supplement: S1 Table — (DOCX) [file pone.0304596.s002.docx]

STable 1: Mixed Methods Appraisal Tool (MMAT) Quality Appraisal

|  | Chamodraka (2008) | De Pretto (2020) | Hulme (1997) | Jones (2015) | MacArthur (2020) | Van Rooji-Peiman (2020) | Wilson (2012) |  | Lam (2017) |
| --- | --- | --- | --- | --- | --- | --- | --- | --- | --- |
| Screening items |  |  |  |  |  |  |  | Screening items |  |
| *S1. Are there clear research questions?* | Yes | Yes | Yes, in the form of an expressed purpose | Yes | Yes | Yes | Yes, in the form of an expressed purpose | *S1. Are there clear research questions?* | Yes, in the form of an expressed purpose |
| *S2. Do the collected data allow to address the research questions?* | Yes | Yes | Yes | Yes | Yes | Yes | Yes | *S2. Do the collected data allow to address the research questions?* | Yes |
| Qualitative studies |  |  |  |  |  |  |  | Mixed method studies |  |
| *1.1. Is the qualitative approach appropriate to answer the research question?* | Yes | Yes | Yes | Yes | Yes | Yes | Yes | *5.1. Is there an adequate rationale for using a mixed methods design to address the research question?* | No, no rationale is provided |
| *1.2. Are the qualitative data collection methods adequate to address the research question?* | Yes, one method was used | No, the data collected were relevant but not fully referent to research questions | Yes, three different and complementary methods were used | Yes, one method was used | No, the extracts were reflective writings that were non-anonymous and that covered relevant topics but were not created in reference to the research questions | Yes, a longitudinal qualitative case study method was used with graphic elicitation | Yes, two complementary methods were used | *5.2. Are the different components of the study effectively integrated to answer the research question?* | No, there was no formal integration |
| *1.3. Are the findings adequately derived from the data?* | Yes, a very thorough description of the analytic approach was provided | No, the analysis is incompletely articulated | Yes, a thorough analytic account is presented, findings are also further explicated in the form of generated propositions (inferences) | Yes, a thorough description of the analytic approach was provided | No, the method of analysis is incompletely explicated | Yes, there was a thorough explanation of the analytic method | No, whilst there are in-depth discussions of methods and processes relevant to data collection, the actual method of analysis is not explicated | *5.3. Are the outputs of the integration of qualitative and quantitative components adequately interpreted?* | No, there was no formal integration to interpret |
| *1.4. Is the interpretation of results sufficiently substantiated by data?* | Yes, illustrative quotes are used very comprehensively | No, there is insufficient presentation of quotes and insufficient linkage between interpretations and data | Yes, illustrative quotes are used very comprehensively | No, whilst illustrative quotes are provided, there are interpretations made at times with no textual evidence | Yes | Yes | No, on some occasions, in-depth interpretations are given without textual evidence | *5.4. Are divergences and inconsistencies between quantitative and qualitative results adequately addressed?* | No, these were not addressed |
| *1.5. Is there coherence between qualitative data sources, collection, analysis and interpretation?* | Yes | Yes, broadly speaking | Yes | Yes | Yes | Yes | Yes | *5.5. Do the different components of the study adhere to the quality criteria of each tradition of the methods involved?* | Yes |
