# Supplementary material for: The light at the end of the tunnel? A systematic review of higher education student experiences of hope
Source: PLoS One. 2024 Jun 17;19(6):e0304596. doi: 10.1371/journal.pone.0304596 (PMC11182537; doi:10.1371/journal.pone.0304596)
Supplement: S2 Table — (DOCX) [file pone.0304596.s003.docx]

STable 2: GRADE-CERQual Evidence Profile and Summary of Qualitative Findings

| Review finding | Studies contributing (First author (date)) | Assessment of methodological limitations | Assessment of coherence | Assessment of adequacy of data | Assessment of relevance | Overall assessment of confidence | Explanation of judgement |
| --- | --- | --- | --- | --- | --- | --- | --- |
| 1. Hope is fundamental: This theme positions hope as fundamental, something of immense importance and an inherent part of the human experience. Students spoke of hope as innate, a fundamental characteristic of being human. Some students connected a sense of hope to religious faith or spirituality. | Chamodraka (2008); Hulme (1997); Jones (2015); MacArthur (2020) | Minor concerns. Two of the studies were of very high quality and different analytic approaches are represented. Hulme’s thesis has concerns about language and the translation of words such as “innate” obscuring meaning. MacArthur’s paper had concerns about how the non-anonymity of participants may have affected the elicitation of data, although this issue did not appear to have prevented participants from describing hope. | Minor concerns. Some uses of the word “innate” in Jones’ thesis may reflect a departure from that outlined in the theme. Moreover, there is some tension between hope construed as innate, versus hope construed as cognitive as emphasised in another theme. | Minor concerns. Different data sources are represented, although half of the studies are not represented here. The data are largely drawn from one study, but this study contained group and individual interviews and diary extracts. Other studies represent interviews and reflective writing. Thus, data do have richness and depth. | Moderate concerns; studies with partial relevance. The data extracts supporting this theme are largely drawn from Hulme’s thesis, which involved students from two private universities, one of which is Christian. Assertions of hope as innate and as a faith-based experience may best reflect the experiences of religious students. | Moderate confidence | Four studies contributed to this conclusion, representing three Western countries. Minor concerns regarding methodological limitations, coherence, and data adequacy. Moderate concerns regarding relevance and aspects of the theme conclusion pertaining to linkage between faith and hope may best apply to students with religious or spiritual beliefs. |
| 1. Hope is the construal of self over time: This theme described how hope underlies self-construal over time. Hope is the means of connecting past and present experiences with the hope-for future self, using the latter to make sense of past experiences and motivate present behavioural change. | Chamodraka (2008); Hulme (1997); Jones (2015); MacArthur (2020); Van Rooji-Peiman (2020) | Minor concerns. The three highest quality studies contributed to this theme, including the single study that used longitudinal data collection. Different analytic approaches are represented. | Minor concerns. The theme is somewhat of an abstraction in that students more rarely identified hope explicitly as the form of their self-construal. However, the interpretation is grounded in textual evidence as provided. | No or very minor concerns. Different data sources are represented. The data describing this conceptualisation of hope have richness and depth and reflect both explicit and implicit connections between self-construals at different points in time. | Minor concerns; partial relevance. The majority of studies were included, but the populations and settings are mostly reflective of students in the West. Although there is some ethnic diversity, the notion of self-construal over time may best reflect the experiences and cognitions of Western people and of more individualistic cultures. | Moderate confidence | Five studies with minor methodological limitations. Data from four countries, but predominantly Western focus. Minor concerns about coherence and adequacy. |
| 1. Hope is goal-directed: This theme identified hope as inherently goal-directed, with goals linked to the pursuit of one’s hope-for future self. The mental invocation of one or more desired goals becomes the focus of hope and, the more specific and realistic the goal, the more it occupies and sustains one’s hopeful thinking. | All | No or very minor concerns. All studies contributed to this theme, including those of very high quality, and therefore the impact of methodological limitations is minimised. | Minor concerns. This finding reflects the data extracted. A small number of quotes reflect students describing hope in broader or more vague terms. However, the identification of hope as relevant to specific goals is very well supported by data from all underlying studies | No or very minor concerns. There was a large number of illustrative quotes from which to draw, additionally with clear examples of varied goals. Different data sources are represented. | No to very minor concerns; direct relevance. All studies were included, thus reflecting some diversity with respect to sociodemographic characteristics and levels of hopefulness. | High confidence | Eight studies, spanning seven countries, with some diversity within participants. Studies include those of high quality. Minor concerns regarding coherence, no concerns regarding data adequacy or relevance. |
| 1. Hope is thinking, doing, and feeling: This theme reflects the conceptualisation of hope as cognitive, behavioural, and affective. Students did identify these components variably, i.e., some students identified hope as primarily cognitive or affective, whereas some emphasised the interplay of these different facets. | All | No or very minor concerns. All studies contributed to this theme, including those of very high quality, and therefore the impact of methodological limitations is minimised. | Moderate concerns. There was more limited evidence of individuals construing hope as having all three components. Moreover, some students described hope clearly as one component only, e.g., that hope is cognitive and not emotional. | Minor concerns. There was textual evidence for the conceptualisation of hope as cognitive, as behavioural, and as emotional. There was more limited data regarding hope as behavioural and/or emotional, i.e., there was more data in favour of hope as cognitive and the data regarding the latter two conceptualisations were a little thinner. | No to very minor concerns; direct relevance. All studies were included, thus reflecting some diversity with respect to sociodemographic characteristics and levels of hopefulness. | Moderate confidence. | Eight studies, spanning seven countries, with some diversity within participants. Studies include those of high quality. Moderate concerns regarding coherence, minor concerns regarding data adequacy, no concerns regarding relevance. |
| 1. Hope is connection: This conceptualisation identifies hope as being built upon relational foundations, including both close relationships and other connections e.g., interactions with university faculty. Mechanisms of influence included promoting a sense of mutuality and mattering, and supporting specific processes such as engagement with one’s identified goals. | Chamodraka (2008); De Pretto (2020); Hulme (1997); Jones (2015); Lam (2017); MacArthur (2020) | Minor concerns. Most studies contributed to this theme, and therefore methodological limitations are largely offset. Different analytic approaches are represented. | Minor concerns. This theme is well supported by the extracted data from the underlying studies. Students spoke explicitly about the relational priming of hope, with evidence also of more implicit linkages between hope and interpersonal relationships and interactions. However, there is uncertainty as to whether hope is best understood as connection versus influenced by connection. | No or very minor concerns. There were a very large number of illustrative quotes from which to draw, which were typically rich in detail. Different data sources are represented. | No to very minor concerns; direct relevance. Most studies are represented, thus reflecting some diversity with respect to sociodemographic characteristics and levels of hopefulness. | Moderate confidence | Eight studies, spanning seven countries, with some diversity within participants. Minor concerns regarding coherence and methodological limitations, no concerns regarding data adequacy or relevance. |
| 1. Hope is resilience: This theme describes how hope confers a sense of resilience, helping students to cope with challenging circumstances and protecting them from the negative impacts of difficulties. | Hulme (1997); Jones (2015); Van Rooji-Peiman (2020) | Minor concerns. Two of the highest quality studies contributed to this theme. Different analytic approaches are represented. | Moderate concerns. The interpretation in this finding is somewhat supported by data from several studies. However, there is some uncertainty as to whether hope is conceived of as resilience versus being related to, but distinct from, resilience. In the following theme, there was reference to the latter position. | Moderate concerns. Different data sources are represented. However, the number of studies and amount of data is more limited than for other themes. The minority of studies are represented here. | Minor concerns; partial relevance. Fewer studies are represented here. The experiences of students in multiple countries are represented, albeit Western in focus. One study did nonetheless focus on participants who had been looked-after children in the Caribbean. This theme is evidenced in studies which purposefully sought to include students who had experienced adversity and those who did not. | Moderate confidence | Three studies contributed to this review, representing three Western countries. Minor concerns regarding relevance and methodological limitations, yet moderate concerns regarding data adequacy and coherence. The resilience-giving properties of hope seem a clear finding, but are captured to some extent in themes 2 and 9. |
| 1. Hope is dynamic and reciprocal: Hope waxes and wanes over time and in response to different life circumstances, and components of hope and other positively-oriented phenomena (e.g., optimism) influence each other in non-recursive feedback loops. | Chamodraka (2008); Hulme (1997); Jones (2015); Lam (2017); Van Rooji-Peiman (2020) | No or very minor concerns. The three highest quality studies contributed to this theme. Different analytic approaches are represented. | Minor concerns. This theme is supported by the underlying studies, albeit with less explicit reference to dynamism. The theme of reciprocity is, in part, about the seemingly self-reinforcing nature of hope and in part about the influence of hope on variables that are overlapping but perhaps distinct. There remains some uncertainty regarding which variables may be construed part of hope versus distinct yet overlapping. | Minor concerns. Although different data sources are represented, there was limited data that explicitly detailed the dynamism of hope. Nonetheless, this dynamism is implied in both data presented and within the construction of hope as non-recursive, i.e., the latter implies a sense of movement and change. There was more data from different sources regarding the reciprocity of hope, i.e. the “circle” metaphor was used in more than one source. | Minor concerns; direct relevance. The majority of but not all studies are represented here. There appears to be representation across different geographical locations and sociodemographic diversity. | Moderate confidence | Five studies contributed to this review, representing five countries. Minor concerns regarding all components, but the theme is nonetheless well-supported by the underlying studies and produced data. |
| 1. Depression is the absence of hope: This theme reflects the identification of depression as reflecting the total absence of hope, and that students experienced depression as connected to a sense of a lost future. | Chamodraka (2008); Hulme (1997); Van Rooji-Peiman (2020) | Minor concerns. All studies that contributed to this theme were of high quality. Different analytic approaches are represented. However, it is not assured that the students who provided data had experienced depression, i.e., studies did not involve a clinical threshold based on the results from a diagnostic interview, for example. | No or very minor concerns. The crux of the theme seems well-supported by the underlying studies, although smaller in number. | Moderate concerns. Although different data sources are represented, this theme does reflect a smaller amount of data from a subset of students from a small number of studies. This likely reflects that a focus on hope in a mental health/problem context was not a given focus of the studies reviewed, although one study explored gains in hope during psychotherapy. | Minor concerns; partial relevance. Three studies are represented here, two of which took place in USA and Canada respectively. This theme is supported by the inclusion of studies which focused on students accessing psychotherapy as well as general/subject-specific student samples. The age and ethnicity of two of the three study samples is unknown. | Moderate confidence | Three studies, representing three Western countries. Minor concerns regarding relevance and methodological limitations, coherence and relevance. Moderate concerns regarding data adequacy with respect to a smaller amount of relevant data. However, linkage to mental health problems was not the given foci of included studies, and the available data do support the theme concept. |
| 1. Hope is a positive, perhaps borne from a negative: Hope was focused on a positive and desired future and the identification of positive goals, and involved positive cognitions and emotions. However, hope was often described as being borne from students observing or experiencing something aversive, either something lacking or traumatic. | All | No or very minor concerns. All studies contributed to this theme, including those of very high quality, and therefore the impact of methodological limitations is minimised. | No or very minor concerns. The crosscutting theme is well supported by the underlying studies. | No or very minor concerns. All studies are represented here, thus drawing data from different sources. | Minor concerns; direct relevance. The “negative” origin of hope is most strongly evident in the accounts of vulnerable studies yet is not knowingly unique to these subsamples. | High confidence | Eight studies, spanning seven countries, with some diversity within participants. Studies include those of high quality. Minor concerns regarding relevance, no concerns regarding data adequacy or coherence. |
