# Supplementary material for: The light at the end of the tunnel? A systematic review of higher education student experiences of hope
Source: PLoS One. 2024 Jun 17;19(6):e0304596. doi: 10.1371/journal.pone.0304596 (PMC11182537; doi:10.1371/journal.pone.0304596)
Supplement: Attachment — Submitted filename: Hope changes.docx [file pone.0304596.s004.docx]

Interesting paper. I have a few general comments and them some more specific ones below.

1—check the sentence length of your sentences. You have many that are extremely long. Please rewrite those that are more than 5 lines long. I use 3 as my rule for a sentence that is too long

2—(Hume, 2020; p >>) not (Hume, 2020) (p. p >>). You have this many times throughout the paper

3—I think it is best to have a concluding sentence rather than a direct quote at the end of paragraphs.

4—The long block quotes should be indented in APA but I am not sure in the Vancourver

The first sentence in the abstract is holding me up. Rather than ‘and’ and ‘or’ can you use a comma the first time and then ‘and’ at the end

The listing of your themes is difficult to parse. Can you reword. The ands ae definitely throwing me off

The way that you state the themes is perfect for this

Set the stem Hope is…. And then list them but watch your ands

Aspinwall and Leaf (2002). Do not add the names again in the parens

On the 3^rd^ page in the second full paragraph there is a sentence that begins with Lay theories affect----please cut the last two words----in addition.

Data extraction

You have an extremely long sentence here. Please break it up into smaller separate sentences.

On page 6 (MMAP) (Houg et al., 2018) APA---- (MMAP; Hough et al., 2018)

Page 7 first lines

Sougth for to full-test------something is wrong

Seems like it may be two?

That first paragraph on page 7 is too long. Please break up the phrases and make them into their own sentence

Page 7--Later you have ( xxx(xxxxx, xxxx) The second one should be a :

Same again later in that paragraph

Most studies involved BOTH……… (towards bottom of page 7)
