# Supplementary material for: The light at the end of the tunnel? A systematic review of higher education student experiences of hope
Source: PLoS One. 2024 Jun 17;19(6):e0304596. doi: 10.1371/journal.pone.0304596 (PMC11182537; doi:10.1371/journal.pone.0304596)
Supplement: Attachment — Submitted filename: Response to Reviewers.docx [file pone.0304596.s005.docx]

We thank the reviewer and editor for their positive and helpful comments and address each in turn.

**Reviewer 1:**

5. Review Comments to the Author

Please use the space provided to explain your answers to the questions above. You may also include additional comments for the author, including concerns about dual publication, research ethics, or publication ethics. (Please upload your review as an attachment if it exceeds 20,000 characters)

Reviewer #1: As an assistant professor working with undergraduate and graduate students in the field of Education of the Deaf and Hard of Hearing at a small university, I found your manuscript particularly relevant and engaging. My role involves extensive interaction with college students, and understanding their experiences, particularly in terms of hope and well-being, has a crucial aspect of my work and even more so post-Covid.

Your systematic review and meta-synthesis are impressively thorough. The methodological approach, including the extensive literature search and careful selection of studies, is admirable. The use of quality appraisal tools like MMAT and GRADE-CERQual further strengthens the reliability of your findings.

Given the qualitative nature of your study, the absence of detailed statistical analysis seems appropriate. The thematic analysis approach you've used effectively synthesizes the data and supports your conclusions in a meaningful way.

The Data Availability Statement in your manuscript is clear and adheres to the principles of transparency and accessibility, essential in research. Additionally, I found no issues regarding dual publication, research ethics, or publication ethics in your paper.

Your research significantly enriches our understanding of student experiences in higher education and is particularly meaningful to those of us dedicated to student education and welfare. Given my background, it would be fascinating to see similar research conducted specifically with Deaf and hard of hearing college students. Such studies could provide deeper insights into the unique experiences and challenges faced by this student population.

The process has been highly informative and a valuable learning experience for me. I've gained new insights into the field of higher education student experiences, which I can apply in my own academic and professional endeavors. This review has not only broadened my understanding of the subject matter but also enhanced my skills in critical analysis and evaluation of research work.

Thank you for the opportunity to review this work.

*Response: We thank the reviewer very kindly for their incredibly positive words, this is so appreciated. We totally agree with the need to explore the experience of hope for vulnerable or marginalised student groups and the groups you mention are a very important example.*

**Editor:**

Interesting paper. I have a few general comments and them some more specific ones below.

*Response: Thank you for your comments, we appreciate these and have responded to requested revisions in turn.*

1—check the sentence length of your sentences. You have many that are extremely long. Please rewrite those that are more than 5 lines long. I use 3 as my rule for a sentence that is too long

*Response: We have reduced these.*

2—(Hume, 2020; p >>) not (Hume, 2020) (p. p >>). You have this many times throughout the paper

*Response: Apologies, this is an artefact of the reference management software, we have corrected this throughout.*

3—I think it is best to have a concluding sentence rather than a direct quote at the end of paragraphs.

*Response: We have added these or otherwise re-structured content to avoid ending with a quote.*

4—The long block quotes should be indented in APA but I am not sure in the Vancourver

*Response: We have indented 40 word plus quotations after checking this is in line with the Vancouver model.*

The first sentence in the abstract is holding me up. Rather than ‘and’ and ‘or’ can you use a comma the first time and then ‘and’ at the end

*Response: We have corrected this.*

The listing of your themes is difficult to parse. Can you reword. The ands ae definitely throwing me off

The way that you state the themes is perfect for this

Set the stem Hope is…. And then list them but watch your ands

*Response: We have corrected this.*

Aspinwall and Leaf (2002). Do not add the names again in the parens

*Response: We have corrected this.*

On the 3^rd^ page in the second full paragraph there is a sentence that begins with Lay theories affect----please cut the last two words----in addition.

*Response: We have removed.*

Data extraction

You have an extremely long sentence here. Please break it up into smaller separate sentences.

*Response: We have split this sentence up.*

On page 6 (MMAP) (Houg et al., 2018) APA---- (MMAP; Hough et al., 2018)

*Response: We have corrected this.*

Page 7 first lines

Sougth for to full-test------something is wrong

Seems like it may be two?

*Response: We have corrected this.*

That first paragraph on page 7 is too long. Please break up the phrases and make them into their own sentence

*Response: We have corrected this.*

Page 7--Later you have ( xxx(xxxxx, xxxx) The second one should be a :

Same again later in that paragraph

*Response: We have corrected this.*

Most studies involved BOTH……… (towards bottom of page 7)

*Response: We have corrected this.*
